# Nonsteroidal Anti‐Inflammatory Drugs and Risk of Gastrointestinal Bleeding: A Systematic Review and Meta‐Analysis

**DOI:** 10.1002/cpt.70054

**Published:** 2025-09-07

**Authors:** Abdelrahman G. Tawfik, Ainhoa Gomez‐Lumbreras, Guilherme Del Fiol, Kensaku Kawamoto, Katy E. Trinkley, Thomas Reese, Aubrey Jones, Daniel C. Malone

**Affiliations:** ^1^ Department of Pharmacotherapy College of Pharmacy, University of Utah Salt Lake City Utah USA; ^2^ Department of Biomedical Informatics, School of Medicine University of Utah Salt Lake City Utah USA; ^3^ School of Medicine University of Colorado Anschutz Medical Campus Aurora Colorado USA; ^4^ Department of Biomedical Informatics Vanderbilt University Medical Center Nashville Tennessee USA

## Abstract

Nonsteroidal anti‐inflammatory drugs (NSAIDs) are widely used for pain and inflammation but are associated with gastrointestinal (GI) bleeding. While this risk is well established, most studies evaluate NSAIDs as a homogenous class, limiting clinical decision‐making based on individual agent safety. This systematic review and meta‐analysis aimed to quantify the risk of GI bleeding associated with individual NSAIDs. We searched PubMed from inception through January 2025 using MeSH and free‐text terms for “gastrointestinal bleeding” and nine commonly used NSAIDs. A random effects meta‐analysis was conducted to estimate the pooled odds ratios (ORs) for GI bleeding, with hazard ratios (HRs) and relative risks (RRs) treated as approximations of ORs due to the rare event nature of GI bleeding. Of 6,711 records screened, 25 studies met the inclusion criteria. Substantial heterogeneity in study design, populations, and outcome ascertainment was observed. Celecoxib was associated with the lowest risk of GI bleeding (OR 1.16, 95% CI: 0.84–1.61). Among non‐selective NSAIDs, ibuprofen had the lowest significant risk (OR 2.28, 95% CI: 1.71–3.03), while ketorolac showed the highest risk (OR 20.67, 95% CI: 14.56–29.34). Other agents, such as piroxicam and meloxicam, also demonstrated significantly elevated risks. The risk of GI bleeding varies widely among individual NSAIDs. Celecoxib appears to have the lowest GI risk, though cardiovascular safety must also be considered. These findings highlight the need for personalized NSAID selection and suggest that NSAIDs should not be treated as a uniform class when assessing bleeding risk.

**Study Highlights**

**WHAT IS THE CURRENT KNOWLEDGE ON THE TOPIC?**

NSAIDs are common drugs that are known to raise the risk of gastrointestinal bleeding. But rather than measuring the risk for individual agents, most studies and guidelines assess NSAIDs as a single class, which restricts patient‐specific risk stratification.

**WHAT QUESTION DID THIS STUDY ADDRESS?**

This study systematically reviewed and synthesized the evidence on GI bleeding risk associated with individual NSAIDs, providing agent‐specific risk estimates based on a meta‐analysis of published studies.

**WHAT DOES THIS STUDY ADD TO OUR KNOWLEDGE?**

Significant differences in GI bleeding risk between NSAIDs are revealed in this review. Agents like piroxicam (OR 9.24), meloxicam (OR 6.85), and ketorolac (OR 20.67) were linked to significantly higher risks, whereas celecoxib showed the lowest risk (OR 1.16). The results highlight the significance of drug‐specific evaluation and challenge the common practice of treating NSAIDs as a homogeneous group.

**HOW MIGHT THIS CHANGE CLINICAL PHARMACOLOGY OR TRANSLATIONAL SCIENCE?**

These results support a more individualized approach to NSAID prescribing. Therapeutic safety may be enhanced by taking drug‐specific GI bleeding risk into account when making clinical decisions, especially for high‐risk patients like elderly patients as elderly and those taking anticoagulants. To improve patient outcomes and reduce adverse events, future prescribing guidelines and predictive tools should stratify NSAIDs based on GI safety profiles rather than generalize across the class.

Nonsteroidal anti‐inflammatory drugs (NSAIDs) are widely used medications that reduce inflammation, alleviate pain, and decrease fever. They manage mild to moderate pain associated with dysmenorrhea, headaches, and musculoskeletal injuries.^1^, ^2^ Additionally, they are prescribed to reduce inflammation in chronic conditions like osteoarthritis and rheumatoid arthritis, where managing inflammation can help maintain joint function and relieve pain.^3^ They work by inhibiting the activity of cyclooxygenase (COX) enzymes, which play a crucial role in synthesizing prostaglandins, which are involved in the inflammatory response.^4^

The use of NSAIDs, both by prescription and over‐the‐counter (OTC), is highly prevalent. Use of NSAIDs among patients in ambulatory care settings has increased over the years, despite national recommendations to minimize use.^5^ For instance, between 2006 and 2016, NSAID use in US ambulatory care settings had very high numbers, with 775.7 million NSAID‐involved visits recorded.^6^ NSAID use was most prevalent among adults aged 46–64 years, females, and White individuals with commercial insurance.^6^ The proportion of outpatient visits when NSAIDs were prescribed rose from 8.1% to 9.6%, highlighting the growing reliance on these medications and the need for continued monitoring of their safety.^6^ Moreover, the OTC availability of NSAIDs such as ibuprofen and naproxen has made them readily accessible to the public. Ubiquitous access has led to a high prevalence of their use. A 2022 survey by Statista reported that 57% of US adults with chronic low back pain had used OTC NSAIDs for pain management, with 45% actively using them at the time of the survey.^7^

The mechanisms underlying NSAID‐induced gastrointestinal (GI) bleeding are multifaceted and primarily involve inhibiting COX enzymes, which play a critical role in synthesizing protective prostaglandins in the gastrointestinal tract.^8^ When NSAIDs inhibit COX‐1, the enzyme responsible for producing protective prostaglandins, the mucosal barrier becomes compromised, leading to increased susceptibility to injury from gastric acid and digestive enzymes. This disruption can lead to mucosal damage, erosions, and ulcers, which may result in bleeding. In addition to COX inhibition, NSAIDs can induce direct damage to the intestinal epithelium by increasing permeability and instability of the epithelial barrier.^9^

The risk of upper GI bleeding can increase up to fourfold in NSAID users, and this risk is compounded when NSAIDs are used in conjunction with other medications, such as selective serotonin reuptake inhibitors (SSRIs).^10^, ^11^, ^12^ However, a significant issue in the existing literature is the relatively small number of studies that have focused on individual NSAIDs^11^; many studies treat the entire class of NSAIDs as a homogeneous category.^13^ Moreover, many studies that report individual agent risks focus on GI complications rather than specifically on GI bleeding.^14^, ^15^ For example, in GI complications, celecoxib has been associated with a relatively modest risk of (RR 1.45; 95% CI: 1.17–1.81), whereas piroxicam (RR 7.43; 95% CI: 5.19–10.63) and ketorolac (RR 11.50; 95% CI: 5.56–23.78) carry substantially higher risks.^14^

Given these gaps in literature, there is a clear need for studies that compile and analyze the GI bleeding risks associated with individual NSAIDs. Such evidence could help healthcare providers make more informed decisions, selecting NSAIDs with the lowest risk profiles for specific patients. This study aims to address that need by conducting a systematic review and meta‐analysis, providing a systematic overview of GI bleeding risk across different NSAIDs to support safer, evidence‐based prescribing.

## METHODS

A meta‐analysis of the GI bleeding risk of NSAIDs was conducted, and the results were reported following the Preferred Reporting Items for Systematic Reviews and Meta‐Analyses (PRISMA) guideline.^16^ The PRISMA 2020 checklist can be found in **Supplemental Material** **S1**.

### Search strategy for exposure and outcomes

Comprehensive literature searches were performed using PubMed, applying both “Gastrointestinal Hemorrhage” as a MeSH term and free‐text terms like “GI bleeding” combined with each NSAID of interest (celecoxib, diclofenac, ibuprofen, indomethacin, ketoprofen, ketorolac, meloxicam, naproxen, and piroxicam), resulting in nine distinct search queries. The search was unrestricted regarding publication date and language, with articles available in PubMed from inception through January 2025 considered for inclusion. To refine the search, we applied PubMed filters to exclude ineligible study types (e.g., editorials, commentaries, and case reports) and limit results to human studies, thereby excluding animal studies. These filters were applied during the initial search process to improve relevance and reduce the burden of manual screening (**Supplemental Material** **S2**).

### Inclusion and exclusion criteria

Evidence from meta‐analyses, randomized controlled trials (RCTs), and observational studies was considered. However, case reports, case series, and non‐quantitative reviews were excluded. If comparisons were made in a study, eligible designs included those comparing NSAID monotherapy to non‐use or placebo. Studies were not required to use placebo controls, but comparisons against non‐use or inactive comparators were necessary for inclusion. Studies without a comparison group were excluded.

Meta‐analyses that pooled randomized trials using only summary‐level data were excluded to avoid double‐counting and ensure independence of included data. In contrast, we retained meta‐analyses based on individual participant data (IPD), which function more as large‐scale secondary analyses rather than traditional meta‐analyses. These IPD‐based studies were considered separately and treated similarly to large datasets due to their analytic depth and transparency.

No restrictions were imposed on the indication for NSAID use. Articles of interest were required to report information on GI bleeding to qualify for inclusion. Studies that mentioned bleeding without specifying the site or all GI complications as a composite measure were excluded. Inclusion criteria also required that reporting of GI bleeding be specific to individual NSAIDs. Studies needed to report quantitative measures of risks such as odds ratios (OR), hazard ratios (HR), relative risks (RR), or event counts.

### Study selection

To assess eligibility, two reviewers (AGL and AGT) independently screened the titles and abstracts identified through the search strategy. The same reviewers independently reviewed full‐text articles of potentially relevant studies. Discrepancies regarding study inclusion were resolved through discussion, with consensus reached in consultation with a third reviewer (DCM) when necessary. The reference lists of included articles were manually screened to identify additional relevant studies, and the “Cited by” function in PubMed was used to capture forward citations. AGL, AGT, and DCM jointly performed data verification and extraction to ensure the accuracy and consistency of key variables, including effect estimates. A cross‐reference check was also conducted to ensure that all relevant studies cited within the included articles were considered for inclusion.

### Data extraction and quality assessment

After finalizing the included studies, two reviewers independently extracted key elements using a standardized data extraction sheet (AGT and AGL). Discrepancies were resolved through discussion and, when necessary, input from a third reviewer (DCM). Extracted variables included study design, population characteristics, NSAID exposure details, outcome definitions (GI bleeding), effect measures (e.g., odds ratios (OR), hazard ratios (HR), relative risks (RR)), confidence intervals, and sample sizes. Individual NSAIDs stratified studies to allow for drug‐specific analysis. When available, adjusted estimates were prioritized. When product‐specific data of risk were not reported, we calculated OR and 95% confidence interval (CI) using standard methods.

The quality of the included observational studies was assessed using the Newcastle–Ottawa Scale (NOS),^17^ which evaluates three domains: selection of study groups, comparability of cohorts, and ascertainment of outcomes. The NOS assigns a maximum score of nine points. Studies receiving a score of six or higher were considered to be of high methodological quality.

### Statistical analysis

A formal meta‐analysis was conducted to pool the risk of GI bleeding associated with each NSAID. A random effects model accounted for between‐study heterogeneity. Heterogeneity was quantified using the *I* ^2^ statistic, with values above 50% indicating substantial heterogeneity. HR and RR were treated as odds ratio OR approximations in meta‐analysis. All analyses were performed using R (version 4.5.0) with the meta package, employing the restricted maximum likelihood (REML) estimator for between‐study variance (τ^2^) and the Hartung‐Knapp‐Sidik‐Jonkman (HKSJ) method to provide adjusted confidence intervals. Additionally, 95% prediction intervals were reported to reflect expected effect ranges in future comparable settings.

To prevent potential double‐counting, we excluded meta‐analyses that synthesized summary‐level data from randomized trials. We also conducted a sensitivity analysis excluding the IPD of randomized controlled trials (RCTs) study to assess its influence on pooled estimates. In addition, we performed a subgroup analysis by study design, comparing results from cohort/nested case–control studies versus case–control studies.

## RESULTS

**Figure 1:**
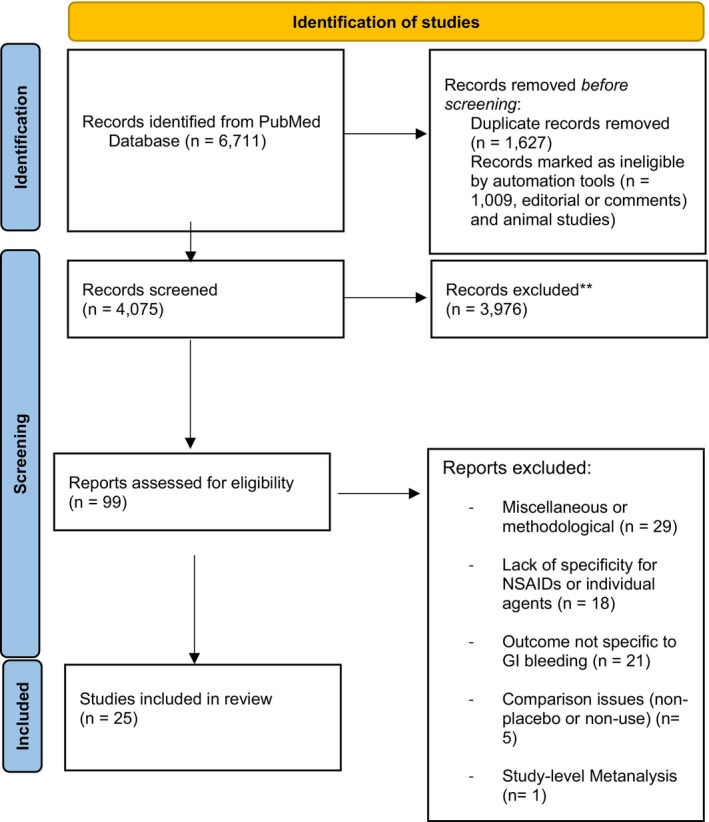
PRISMA flow diagram.

A total of 6,711 records were identified through PubMed searches. Of these, 1,627 were excluded as duplicates, 576 records were excluded due to ineligible study type (such as editorials and commentaries), and 433 records were excluded due to being animal studies, leaving a total of 4,075 records for further screening. During the initial screening with titles and abstracts, 3,976 records were excluded for various reasons, including irrelevance to NSAID‐associated GI bleeding, insufficient data, or methodological limitations. A detailed full‐text review of 99 reports was conducted, excluding an additional 73 reports. Among these, 29 studies were excluded due to methodological issues, 18 studies lacked specificity regarding NSAIDs or individual agents, 21 did not focus on gastrointestinal bleeding as an outcome, and 5 had inappropriate comparison groups (e.g., non‐placebo or non‐control studies). See **Figure** **1** for the PRISMA diagram.

A total of 25 studies met the inclusion criteria,^18^, ^19^, ^20^, ^21^, ^22^, ^23^, ^24^, ^25^, ^26^, ^27^, ^28^, ^29^, ^30^, ^31^, ^32^, ^33^, ^34^, ^35^, ^36^, ^37^, ^38^, ^39^, ^40^, ^41^, ^42^ consisting of 19 case–control studies,^18^, ^20^, ^22^, ^23^, ^24^, ^25^, ^26^, ^27^, ^28^, ^29^, ^30^, ^33^, ^34^, ^36^, ^37^, ^38^, ^39^, ^41^, ^42^ 4 cohort studies,^19^, ^32^, ^35^, ^40^ and 2 IPD meta‐analyses^21^, ^31^ **Table** **1** provides an overview of the selected studies that estimate the risk of GI bleeding for each specific NSAID. To prevent double counting, we confirmed that the three primary studies included in Lewis *et al*. were not duplicated among the other studies. Moreover, Essex *et al*. was excluded because it is a summary‐level data meta‐analysis.^43^

**Table 1:** Characteristics of included studies evaluating the risk of gastrointestinal bleeding associated with individual NSAIDs

Study	Study design	Country	Data source/setting	Study period and/or duration	Exposure definition	Adjustment methods and covariates	Gastrointestinal bleeding identification method	Total sample size (Cases/control)^a^	Population characteristics		
									Age	Female (%)	Race
Alexander 1985^18^	Case–control	United Kingdom	The Hereford Hospital	1977 to 1980	The use of any analgesic or anti‐rheumatic drugs was recorded	Unadjusted analysis: no covariates reported	Diagnosis Code: ICD‐8	1,878 (93/1785)	75 years of age	NA	NA
Anderson 2020^19^	Retrospective cohort	USA	Electronic medical records from a single academic medical center with an annual ED census of ~77,000	January 2017 to December 2017	A single dose of parenteral ketorolac administered in the Emergency Department	Unadjusted logistic regression: no covariates included due to limited events	Diagnosis Code: ICD‐10	480 (120/360)	72.2% (5.8%) were 65 years of age or older	54.2% in cases and 59.2% in controls	80% white
Battistella 2005^20^	Nested case–control	Canada	Ontario Drug Benefit Program Canadian Institute for Health Information Discharge Abstract Database Ontario Registered Persons Database Ontario	April 2000 to March 2001	Prescription for an NSAID in the 90 days before the index date	Logistic regression: adjusted for other NSAID/COX‐2 use, age, sex, comorbidities, prior GI bleeding, number of medications, ICU admission, renal function, and PPI use	Diagnosis Code: ICD‐9	1,798 (361/1,437)	Cases: 79.8 (6.9) years of age Controls: 77.6 (5.5) years of age	51.1% in cases and 51.2 in controls	NA
Begaud 1992^41^	Case–control	France	Hospital of Bordeaux and Toulouse	February 1987 to December 1988	OTC or prescribed NSAIDs prior 4 months to the index date	Logistic regression: adjusted for age, sex, number of drugs used in the 7 days before the index day, ulcer history, alcohol, smoking, analgesic or NSAID use	Endoscopic confirmation	271 (79/192)	NA	NA	NA
Blot 2000^42^	Case–control	USA	American College of Gastroenterology survey	1995	OTC or prescribed NSAIDs 1 week prior to the index date	Logistic regression; adjusted for age, sex, alcohol use, dyspepsia, prior GI bleeding, corticosteroid/anticoagulant use, and other analgesic use	Hospitalized patients for bleeding	1,217 (627 /590)	Cases: 23.9% were 50–64 years of age 44.8% were 65 years of age or older Controls: 27.6% were 50–64 years of age 33.2% were 65 years of age or older	37% in cases and 50.3 in controls	
Bhala 2013^21^	Meta‐analysis of individual participant data	Varies	Meta‐analyses of 280 trials of NSAIDs vs. placebo	NA	Varies	NA – Individual Participant Data (IPD) meta‐analysis; centralized analysis across trials	Clinical trial reporting	88,367 (NA)	60.1 (12.4) years of age	59% in both cases and controls	82% Caucasian
de Abajo 2013^22^	Nested Case–control	Spain	BIFAP Database (Base de datos para la Investigación Farmacoepidemiológica en Atención Primaria)	January 2001 to June 2005	Current users (≤30 days before the index date), past users (31–365 days prior), and non‐users.	Adjusted for age, sex, calendar year, smoking, history of peptic ulcer, number of GP visits (as proxy for comorbidity), and concomitant use of medications associated with UGI	ICPC codes + string text algorithm to identify its gastrointestinal origin and active hemorrhage in the linked free‐text notes	11,193 (1,193/10,000)	65.0 (13.2) years of age in cases and 64.3 (13.2) in controls	36.2in cases and 35.4 in controls	
Garcia 1994^23^	Nested case–control	United Kingdom	United Kingdom General Practice Research Database (GPRD)	January 1990 to February 1993	Use of any NSAIDs during the month before admission.	Logistic regression, adjusted for age, sex, prior peptic ulcer disease, smoking, alcohol use, oral corticosteroids, anticoagulants, aspirin use, and NSAID exposure characteristics (including dose, duration, multiple use, and individual NSAID types)	Computer identification OXMIS code for UGIB, then manually reviewed	9,879 (862/9,017)	NA	NA	NA
Garcia 1998^24^	Case–control	Italy	The region of Friuli‐Venezia Giulia (F‐VG) database	January 1991 to June 1995	Current User: (1–30 days) Past User: (31–60 days) Distant Past User: (61–150 days) Non‐use (> 150 days)	Logistic regression was used, adjusting for NSAID exposure characteristics (use status, dose, duration, multiple use, specific agents, route), demographic factors (age, sex, calendar year), ulcer history, comorbidities co‐medications (aspirin, corticosteroids, antiplatelets, anticoagulants, acid suppressants), and morbidity markers (e.g., digoxin, β‐agonists, insulin).”	Diagnosis Code: ICD‐9	18,837 (907/17,930)	62.3% above 60 years of age in cases and 34.5% above 60 years of age in controls	36.8% of cases and 53.1% of controls	NA
García 2001^25^	Nested case–control	United Kingdom	United Kingdom General Practice Research Database (GPRD)	April 1993 and October 1998	Non‐use (>180 days) Current (0–30 days) Recent (31–90 days) Past (91–180 days)	Logistic regression models were used to estimate the relative risks, adjusting for age, sex, calendar year, smoking, antecedents of upper gastrointestinal disorders, and the use of steroids, anticoagulants, non‐aspirin NSAIDs, acetaminophen, H2 receptor antagonists, omeprazole, misoprostol, and aspirin	Chart review	13,605 (2,105/11,500)	40–79 years of age	NA	NA
Gutthann 1997^26^	Nested case–control	Canada	Saskatchewan Department of Health. Saskatchewan	January 1982‐ to December 1986	Non‐use (> 150 days) Current (0–30 days) Recent (31–60 days) Past (61–150 days)	Logistic regression; adjusted for calendar year, age, sex, and exposure to NSAIDs, oral corticosteroids, and prescription aspirin	Diagnosis codes (ICD‐9) plus chart review	10,705 (1,158/9,547)	40–79 years of age	NA	NA
Lanas 2003^27^	Case–control	Spain	Hospitals	November 1995 to February 1998	Use of NSAIDs during the week preceding hospital admission.	Logistic regression; adjusted for age, sex, previous gastrointestinal bleeding, ulcer history, cardiovascular disease, cerebrovascular disease, use of antisecretory drugs, nitro vasodilators, and analgesics	Endoscopy or other diagnostic procedures	3,353 (1,122/2,231)	65.1 (16.6) years of age in cases and 65.2 (16.7) in controls	30.7% of cases and 30.8% in controls	NA
Lanas 2006^28^	Case–control	Spain	Hospitals from the National Health System.	2001 to 2004	Current (0–7 days) Past (>8 days) non‐use: no reported use	Logistic regression model included age, sex, calendar semester, ulcer history, nitrates, anticoagulants, antiplatelets, acid‐suppressing drugs, coxib, and aspirin use	Observation: (hematemesis or melena), confirmed by hospital personnel through an endoscopic diagnosis	8,309 (2,777/5,532)	61 years of age (no measure of variation provided)	NA	NA
Lanas 2015^29^	Case–control	Spain	Hospitals integrated within the Spanish Association of Gastroenterology and the Biomedical Investigation Network Center of Hepatic and Digestive Diseases (CIBERehd)	2009 to mid‐2013	Current (0–7 days) Past (>8 days) non‐use: no reported use	Logistic regression included age, sex, hospital, calendar semester, gastrointestinal history, smoking status, oral anticoagulants, antiplatelet agents, acid‐suppressing drugs, NSAIDs, COX‐2 inhibitors, and aspirin.”	Endoscopy or other diagnostic procedures	2,016 (1,008/1,008)	66.6 (16.0) years of age among cases and 65.6 (15.5) among controls	NA	NA
Laporte 2004^30^	Case–control	Spain and Italy	Hospitals in Spain and Italy	Spain: September 1998 to December 2001 Italy: November 1999 to December 2001	Use of NSAIDs and analgesics within 7 days before the index date	Logistic regression was used, adjusting for center, admission date, sex, age, peptic ulcer history, diabetes, heart failure, smoking, alcohol, individual NSAIDs and analgesics, dose and duration of NSAID/acetaminophen use, antiplatelet drugs, acid‐suppressing drugs (antacids, H2‐blockers, PPIs, misoprostol, sucralfate), nitrates, topical NSAIDs, calcium channel blockers, and SSRIs. Corticosteroids and anticoagulants were excluded	Diagnosis codes or mixed lesions with an endoscopic procedure	10,006 (2,813/7,193)	NA	NA	NA
Lewis 2002^31^	A meta‐analysis based on individual patients for three case–control studies (IPD)	Varies	Conducted in Catalonia, British, and Sweden.	NA	Use of NSAIDs and analgesics within 7 days before the index date	Logistic regression Adjusted for: Age, sex, study site, smoking, upper gastrointestinal (UGI) history, aspirin use, paracetamol use, anticoagulant use, individual NSAIDs (including dose and duration), and concurrent NSAID use	Observation confirmed by endoscopy, radiology, or surgery	7,632 (2,281/5,351)	Britian: 74 (7.8) Catalan: 58 (17.1) Sweden: 64 (15.2)	Britain: 44.2%. Catalan: 31.3%. Sweden:46.9%	NA
Mamdani 2002^32^	Retrospective cohort	Canada	Ontario Drug Benefit Program Canadian Institute for Health Information Discharge Abstract Database Ontario Health Insurance Plan Ontario Registered Persons Database Ontario.	April 2000 to March 2001	At least two successive prescriptions with coverage for at least 30 days	Cox proportional hazards model Adjusted for: Age, sex, long‐term care, low‐income status, prior hospitalizations, prior GI procedures, number of medications in the prior year, narcotic or gastroprotective use (past 180 days), and concurrent use of aspirin, anticoagulants, antiplatelets, antidiabetic agents, antirheumatics, glucocorticoids, and gastroprotective agents	Diagnosis codes: ICD‐9	118,908 (18,908/100,000)	76.5 (6.8) years of age	70%	NA
Nobili 1992^33^	Case–control	Italy	Network within the Italian National Health Service. Northern Italy	January 1987 to December 1988	Endoscopically confirmed primary hematemesis and/or melaena, or primary diagnosis of acute upper gastrointestinal bleeding	Logistic regression Adjusted for: Sex, age, cigarette smoking, symptoms before admission and exposure to aspirin, pyrazalone derivatives, paracetamol, piroxicam, diclofenac, aryl alkanoic acid derivatives (naproxen and ibuprofen), and indomethacin	Endoscopy	1,764 (441/1,323)	42.8% above 65 years of age	36%	NA
Nørgård 2004^34^	Case–control	Denmark	Danish county of North Jutland	January 2000 to December 2002	Use of celecoxib during the 30 days preceding the date of hospital admission.	Logistic regression adjusted for: discharge diagnoses of alcoholism, esophagitis, gastritis, duodenitis or Mallory–Weiss lesions, comorbidity index, age, sex, previous non‐bleeding ulcer diagnosis (0–2 years, >2 years, none), and prescriptions for oral anticoagulants, low‐dose aspirin, high‐dose aspirin, glucocorticoids, selective serotonin reuptake inhibitors, PPIs, and H_2_‐antagonists	Diagnosis codes: ICD‐8 and ICD‐10	3,686 (780/2,906)	66.8 (15.9) in cases and 72.5 (13.5) in controls	42.9% of cases 46.9% of controls	NA
Rahme 2007^35^	Retrospective cohort	Canada	Healthcare records. Quebec, Canada	April 1999 to December 2002	The number of days supplied from the database plus a 25% grace period.	Cox regression with time‐dependent exposure; Adjusted for: age, sex, ischemic heart disease, heart failure, renal failure, chronic obstructive pulmonary disease, rheumatoid arthritis, osteoarthritis, anemia, alcohol or drug abuse, gastric ulcers, prescriptions for antihypertensives, lipid‐lowering agents, antidiabetics, vasodilators, gastroprotective agents, corticosteroids, anticoagulants, and chest pain	Diagnosis codes: ICD‐9	240,842 (81,932/158,910)	≥65 years	67.2% of cases and 63.2% of controls	NA
Sakamoto 2006^36^	Case–control	Japan	14 hospitals in Japan	September 2002 to December 2004	Use of NSAIDs during the 7 days before the index day.	Logistic regression Adjusted for: Age, sex, region, alcohol consumption, caffeine intake, smoking status, and history of gastric or duodenal ulcer	Observation plus endoscopy	522 (175/347)	52% above 60 years of age for cases and 51% in controls	25% of cases and 26% of controls	NA
Savage 1993^37^	Case–control	New Zealand	Hospitals Christchurch.	NA	Use of non‐aspirin NSAIDs during the week before admission.	Logistic regression Adjusted for: other NSAIDs, aspirin, paracetamol, alcohol, and smoking	Chart review	973 (NA)	19–98 years.	44.5%	NA
Somerville 1986^38^	Case–control	United Kingdom	Nottingham University and City Hospitals.	April 1983 to March 1985	The use of NSAIDs was recorded.	Unadjusted analysis: no covariates reported	Chart review or endoscopy	437 (230/207)	≥60 years	NA	NA
Udd 2007^39^	Case–control	Finland	Central Finland Central Hospital and Kuopio University Hospital	1994 to 1996	Regular or daily NSAID use within 2 weeks before admission.	Logistic regression Adjusted for: Age, sex, history of upper gastrointestinal bleeding, history of gastric or duodenal ulcer, smoking (categorized), alcohol consumption (categorized), NSAID type and dose (ASA, ibuprofen, ketoprofen, naproxen, diclofenac, indomethacin), duration of NSAID use, aspirin for warfarin use, and Helicobacter pylori infection	Endoscopy	188 (94/94)	63.7 (13.5) in cases and 63.8 (12.7) in controls	37.2%	NA
Wan Ghazali 2021^40^	Retrospective cohort	Malaysia	Hospital Raja Perempuan Zainab II. Kota Bharu	January to December 2018	NA	Logistic regression Adjusted for: Age, diclofenac use, and end‐stage renal failure (ESRF); smoking, hypertension, PPI use, and creatinine clearance	Endoscopy	403 (29/374)	67.5 (11.8) in cases and 54.8 (16.3) in controls	3.4% of cases and 11.5% of controls	93.1% Malay in cases and 97.3% in controls

GIB, Gastrointestinal bleeding; ICD, International classification of disease; ICPC, International classification of primary Care; IPD, Individual Participant Data; NA, Not available; NSAID, Non‐steroidal anti‐inflammatory drug; OXMIS, Oxford medical information systems; PPI, Proton Pump Inhibitor; SMQ, Standardized MedDRA Queries; SSRI, Selective Serotonin Reuptake Inhibitors; UGIB, Upper gastrointestinal bleeding.

^**a**^ For cohort studies, the bracketed number is for exposed and non‐exposed individuals.

All included studies were assessed for quality using the Newcastle–Ottawa Scale (NOS), and all received a score of 6 or higher, indicating moderate to high methodological quality. Among the case–control studies, Savage *et al*. was the only study to receive the maximum score of 9.^37^ Most case–control studies lost points on the “Selection of Controls” domain, as they selected hospital‐based rather than community‐based controls, which may introduce selection bias. In the cohort studies, quality was generally high, although two studies had limitations in the representativeness of the exposed cohort. Details on NOS ratings for each study are available in **Supplemental Material** **S3**.^19^, ^40^

### Time and study setting

The selected studies include data collected from 1977 (Alexander *et al*.)^18^ to 2018 (Wan Ghazali *et al*.).^40^ All studies included at least 1 year of data, with several spanning multiple years. Only two studies had a timeline exceeding 5 years: Gutthann *et al*. spanned 5 years from January 1982 to December 1986^26^ and Garcia *et al*. had a duration of 5.5 years from April 1993 to October 1998.^25^ While most of the studies were conducted in Europe or North America,^18^, ^19^, ^20^, ^21^, ^22^, ^23^, ^24^, ^25^, ^26^, ^27^, ^28^, ^29^, ^30^, ^31^, ^32^, ^33^, ^34^, ^35^, ^37^, ^38^, ^39^, ^41^, ^42^ two exceptions were Sakamoto *et al*. conducted in Japan and Wan Ghazali *et al*. using data from Malaysia.^36^, ^40^

### Population

Several studies examined the interaction between NSAIDs and anticoagulants in the context of GI bleeding.^19^, ^20^, ^28^, ^29^, ^39^ Anderson *et al*. did not specifically focus on patients receiving direct‐acting oral anticoagulants, but they noted that anticoagulant use including warfarin, apixaban, edoxaban, rivaroxaban, dabigatran, enoxaparin, fondaparinux, and subcutaneous heparin was balanced between‐study groups.^19^ Battistella *et al*. highlighted the risk of upper GI bleeding in a cohort of 98,821 elderly patients on warfarin, finding an increased risk when combined with non‐selective NSAIDs.^20^ Lanas *et al*. reported that GI bleeding cases were more likely to have used antiplatelet agents, particularly clopidogrel, than controls.^29^ Similarly, Lanas *et al*. found a higher prevalence of antiplatelet agents (e.g., clopidogrel and ticlopidine) in cases.^28^ They identified a dose‐dependent relationship between warfarin and an elevated risk of upper GI bleeding.^28^ Udd *et al*. observed that 7.2% of cases used warfarin, but no controls were receiving warfarin.^39^

Most of the studies focused on elderly populations; however, eight did not report the participants’ ages.^18^, ^23^, ^25^, ^26^, ^30^, ^35^, ^37^, ^38^ Similarly, another eight studies did not provide information on the distribution of participants by sex.^18^, ^23^, ^25^, ^26^, ^28^, ^29^, ^30^, ^38^, ^41^ Notably, race was underreported in most studies, except for a few studies.^19^, ^21^, ^40^ For instance, Anderson *et al*. noted that 80% of participants were White.^19^ A similar percentage was found in the analysis by Bhala *et al*. (82%). On the other hand, Wan Ghazali *et al*. reported that 93% of cases and 97% of controls were Malay.^40^

Several studies employed direct data collection methods, including interviews or structured questionnaires, to gather detailed participant information.^27^, ^28^, ^33^, ^37^, ^38^, ^42^ For instance, Lanas *et al*. (2003) and Lanas *et al*. (2006) collected data through interviews within 48 hours of admission, supplemented by family input and prescription reviews.^27^, ^28^ Similarly, Nobili *et al*. trained hospital staff to conduct face‐to‐face interviews using a structured questionnaire to capture drug exposure history and demographic details.^33^ Savage *et al*. and Somerville *et al*. also relied on interviews to assess drug use, verifying patient recall against medical records where possible.^37^, ^38^ Blot *et al*. used the American College of Gastroenterology survey data to assess NSAID use and bleeding outcomes.^42^

### Exposure definition

The studies investigating GI bleeding risks associated with NSAID use utilized diverse exposure definitions, which can be grouped into distinct categories based on their methodologies. Several studies focused on NSAID or analgesic use within a short, specific timeframe, often in the days or weeks preceding the index event. For instance, Lanas *et al*. (2003, 2006, 2015), Laporte *et al*., Lewis *et al*., Sakamoto *et al*., Savage *et al*., and Blot *et al*. defined exposure as NSAID or analgesic use within 7 days before admission or the index date.^27^, ^28^, ^29^, ^30^, ^31^, ^36^, ^37^, ^42^ This approach was designed to capture acute drug effects contributing to GI bleeding.

Some studies categorized NSAID use into current, recent, past, or non‐use groups over extended periods.^22^, ^24^, ^25^, ^26^ Garcia *et al*. (1998, 2001), Gutthann *et al*., and de Abajo *et al*. applied such classifications, examining usage patterns ranging from 1 to 30 days (current use) to over 150 days (non‐use).^22^, ^24^, ^25^, ^26^ Similarly, Battistella *et al*., Mamdani *et al*., and Nørgård *et al*. specified drug use within a defined period, such as 30 or 90 days before the event.^20^, ^32^, ^34^ In contrast, Rahme *et al*. defined NSAID exposure as the number of days supplied plus a 25% grace period, reflecting a focus on the cumulative supply of drugs.^35^ Distinct from these was Anderson *et al*., who uniquely investigated the effects of a single parenteral dose of ketorolac administered in an emergency department setting.^19^

### Outcome definition

The methods used to identify GI bleeding varied across the included studies, reflecting differences in design, data sources, and clinical context. A large proportion of observational studies identified GIB using diagnosis codes, including ICD‐8, ICD‐9, ICD‐10, or ICPC systems^18^, ^19^, ^20^, ^24^, ^32^, ^34^, ^35^ Some of these studies enhanced coding‐based identification with manual chart reviews, as seen in Garcia 1994^23^ and Gutthann.^26^

Several hospital‐based or case–control studies confirmed GIB through endoscopic findings.^27^, ^29^, ^33^, ^39^, ^40^, ^41^ Other studies used combined methods, such as endoscopy, radiology, or surgery,^30^, ^31^ or relied on clinical observation confirmed by hospital personnel, particularly in acute care settings.^28^, ^36^

In randomized controlled trials and meta‐analyses of trial data, bleeding events were identified using standardized clinical definitions or MedDRA queries, covering a broad range of GI hemorrhage terms.^21^ A few studies also incorporated algorithm processing or free‐text searches within electronic health records to identify GI bleeding more accurately.^22^

This heterogeneity in bleeding identification—from administrative coding to procedure‐confirmed diagnoses—may contribute to differences in reported risk estimates across studies and is a source of potential methodological variability in the pooled analysis.

### Statistical adjustment and risk factor inclusion

Most employed multivariable logistic regression or Cox regression models to adjust for potential confounding.^20^, ^22^, ^23^, ^24^, ^25^, ^26^, ^27^, ^28^, ^29^, ^30^, ^31^, ^32^, ^33^, ^34^, ^35^, ^36^, ^37^, ^39^, ^40^, ^41^, ^42^ In contrast, some studies provided unadjusted analyses, including Alexander *et al*.,^18^ Anderson *et al*,^19^ and Somerville *et al*.^38^

While age and sex were adjusted in nearly all studies (84%),^20^, ^22^, ^23^, ^24^, ^25^, ^26^, ^27^, ^28^, ^29^, ^30^, ^31^, ^32^, ^33^, ^34^, ^35^, ^36^, ^37^, ^39^, ^40^, ^41^, ^42^ history of peptic ulcer or prior GI bleeding was a common covariate (68%) as well.^20^, ^22^, ^23^, ^24^, ^25^, ^27^, ^28^, ^29^, ^30^, ^31^, ^32^, ^34^, ^35^, ^36^, ^39^, ^41^, ^42^ Medication products were accounted for in nearly all multivariable analyses.^20^, ^22^, ^23^, ^24^, ^25^, ^26^, ^27^, ^28^, ^29^, ^30^, ^31^, ^32^, ^33^, ^34^, ^35^, ^37^, ^39^, ^40^, ^41^, ^42^ However, the specific medications included in the adjustment models varied across studies. See **Table** **1**.

### Risk estimate and meta‐analysis

**Figure 2:**
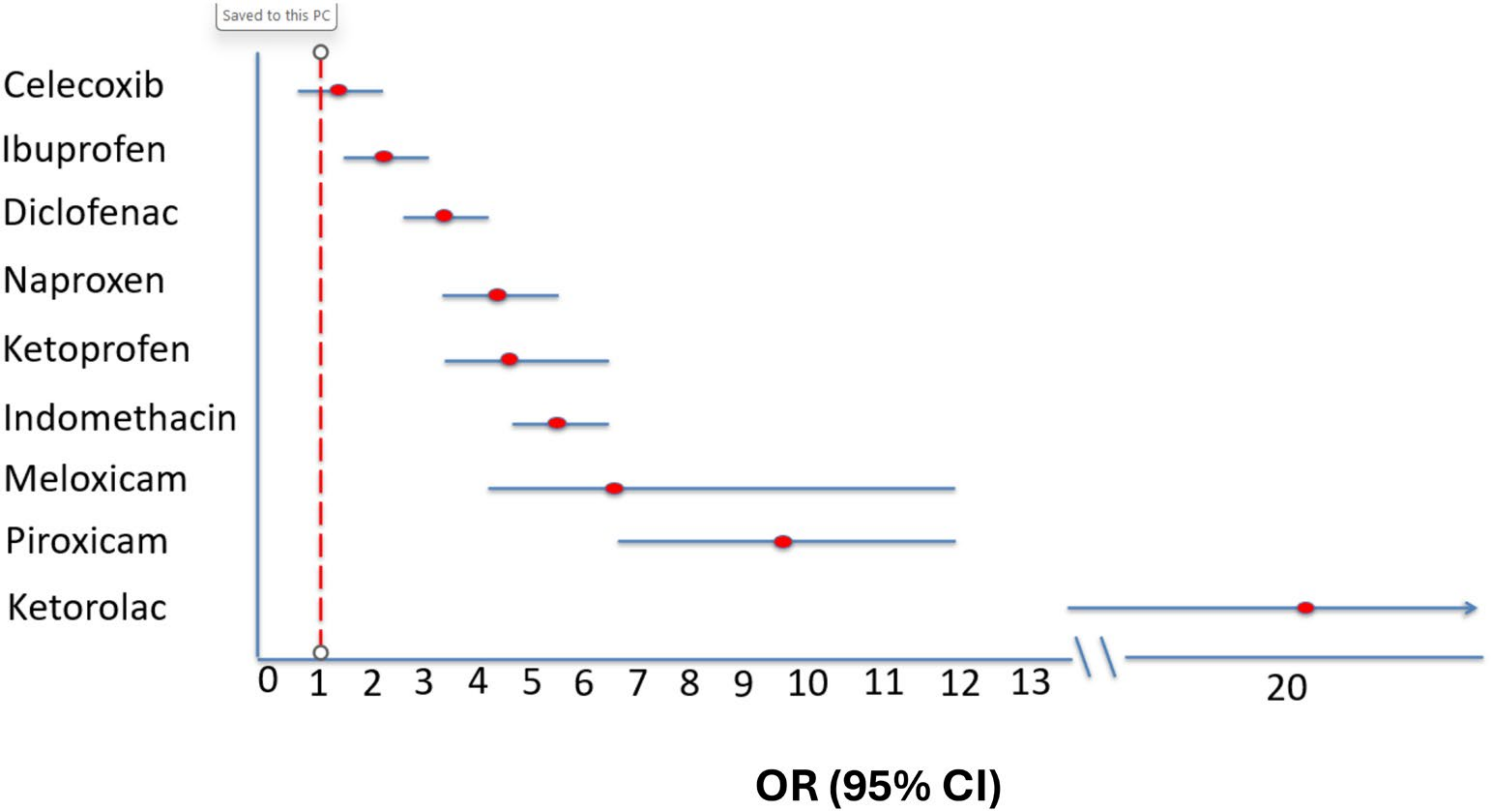
Overall results from meta‐analysis.

**Figure 3:**
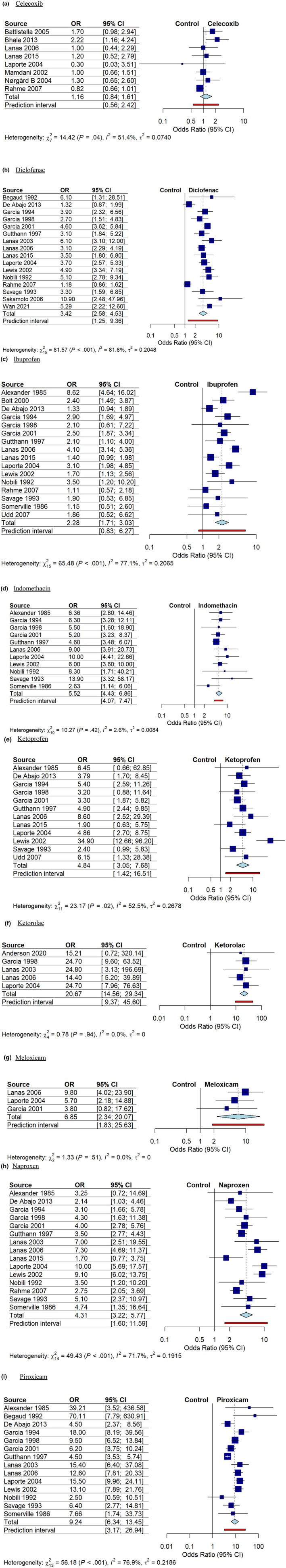
Risk of gastrointestinal bleeding by unique nonsteroidal anti‐inflammatory drugs.

The results of the random effects meta‐analysis for all NSAIDs are shown in **Figure** **2**, with **Figure** **3** providing details for each NSAID. Celecoxib, a selective COX‐2 inhibitor, was not associated with an increase in the risk of GI bleeding with a pooled OR of 1.16 (95% CI: 0.84: 1.61, prediction interval 0.56: 2.42). In a sensitivity analysis excluding the Bhala *et al*. study (an IPD meta‐analysis), the pooled estimate for celecoxib remained non‐significant and slightly attenuated, with an OR of 1.04 (95% CI: 0.78–1.38), and heterogeneity was reduced from 51.4% to 26.4% (**Figure** **S1**).

Ibuprofen, a commonly used non‐selective NSAID, demonstrated the lowest significant risk among all non‐selective NSAIDs, with an OR of 2.28 (95% CI: 1.71–3.03) and a prediction interval of 0.83 to 6.27. Diclofenac showed a moderate increase in risk (OR 3.42; 95% CI: 2.58–4.53; prediction interval: 1.25–9.36). Naproxen was associated with a pooled OR of 4.31 (95% CI: 3.22–5.77) and a prediction interval of 1.60–11.59. Ketoprofen and indomethacin had higher risks, with ORs of 4.84 (95% CI: 3.05–7.68) and 5.52 (95% CI: 4.43–6.86), and prediction intervals of 1.42–16.51 and 4.07–7.47, respectively.

Meloxicam, a partially selective COX‐2 inhibitor, was associated with a pooled OR of 6.85 (95% CI: 2.34–20.07) and a wide prediction interval (1.83–25.63). Piroxicam also showed a markedly elevated risk (OR 9.24; 95% CI: 6.34–13.45; prediction interval: 3.17–26.94). Ketorolac demonstrated the highest risk of GI bleeding, with a pooled OR of 20.67 (95% CI: 14.56–29.34) and a prediction interval of 9.37–45.60.

Evidence of heterogeneity across the studies varied by specific agent. Diclofenac, ibuprofen, naproxen, and piroxicam had *I* ^2^ values greater than 65%. On the other hand, results for celecoxib, indomethacin, ketoprofen, ketorolac, and meloxicam suggested low or modest heterogeneity with *I* ^2^ below 50%.

When stratified by study design, pooled estimates tended to be higher with case–control studies than cohort studies, but not statistically different (*P* > 0.05). The only exception was naproxen (*P* = 0.007). See **Supplemental Material** **S5**.

## DISCUSSION

This review and meta‐analysis provide an estimate of the risk of GIB associated with individual NSAIDs. This meta‐analysis provides estimates of the GI bleeding risk for individual NSAIDs, indicating significant variability in the odds of a bleeding event. Celecoxib was associated with no elevated risk of GI bleeding, highlighting its relative safety. Among non‐selective NSAIDs, ibuprofen, diclofenac, and naproxen appeared to increase the risk at least 2‐fold. In contrast, ketoprofen, indomethacin, and piroxicam were linked to significantly higher risks of GI bleeding than other NSAIDs. Notably, ketorolac had over a 20‐fold increase in risk of GIB compared to no treatment, underscoring its potential for increased GIB.

There was substantial heterogeneity across the included studies regarding population characteristics, exposure definitions, and outcome ascertainment. Many studies focused on adult or elderly populations. However, key demographic details like age, sex, and race were frequently underreported, limiting our ability to assess subgroup effects across populations. Exposure definitions varied across publications with some studies classifying NSAID use as current or recent based on prescription timing (e.g., within 7–30 days before bleeding); others used self‐reported data or cumulative supply duration. GIB outcomes were captured differently—some studies relied on administrative codes (i.e., ICD‐9 or ICD‐10), others used endoscopy‐confirmed diagnoses, or chart reviews. This variability in outcome definitions may partly explain differences in reported risk estimates.

These findings highlight the importance of tailoring NSAID selection to individual patient risk profiles to balance therapeutic benefits and GI bleeding risk. High‐risk populations, such as the elderly, those with a history of previous GI bleeding or ulcers, and patients on anticoagulants, require careful evaluation and monitoring when considering adding NSAIDs to the treatment regimen.^44^, ^45^, ^46^, ^47^, ^48^ For patients where traditional NSAIDs pose unacceptable risks, COX‐2 inhibitors like celecoxib offer a viable alternative with a better safety profile. However, individual risk–benefit assessments should still guide their use, including the patient’s cardiovascular risk and overall clinical judgment.^49^, ^50^

Using medications to reduce GIB like proton pump inhibitors (PPIs) can further mitigate risks.^51^, ^52^ Moreover, non‐pharmacological pain management approaches, such as physical therapy or acupuncture, may be considered as an alternative to or reduce the use of NSAIDs.^53^, ^54^, ^55^ For localized pain, topical NSAIDs like diclofenac gel may offer effective relief, lower systemic absorption, and reduce gastrointestinal risks.^56^

In this review, GI bleeding was the primary outcome of interest. However, many studies have assessed the overall risk of GI complications, rather than specifically focusing on bleeding, for individual NSAIDs.^14^, ^15^, ^57^ Conversely, some studies have examined the combined effect of NSAIDs as a single class with anticoagulants, comparing this combination to anticoagulant use alone.^13^, ^58^ When treated as a single therapeutic class, the risk of GI bleeding has been reported to range from 1.40 (95% CI: 0.80–2.44) to 2.18 (95% CI: 1.02–4.69).^13^, ^58^ This evidence supports our findings that treating NSAIDs as a single therapeutic class may lead to an underestimation of the risk associated with specific agents and is a missed opportunity to provide more patient‐tailored treatment guidance.

Other studies compared the risk of GI bleeding when NSAIDs are used in combination with other medications known to increase the risk of bleeding, such as anticoagulants and SSRIs.^10^, ^12^ While this approach provides valuable insights into the synergistic effect of NSAIDs with other therapies, the reports commonly group NSAIDs together and make it challenging to evaluate unique product combinations. Differentiating the risks associated with specific NSAIDs is crucial for more precise clinical decision‐making, particularly in patients with concomitant anticoagulant therapy or other GI bleeding factors.

For all agents except celecoxib, case–control studies consistently produced higher odds ratios than cohort or nested designs. This pattern likely reflects methodological differences: traditional case–control studies are more susceptible to selection and recall bias, as exposure is often ascertained retrospectively and may be differentially recalled or recorded between cases and controls.^59^ However, for rare outcomes such as GIB, high‐quality case–control studies remain a robust and efficient approach for estimating risk.^60^

A key strength of this study lies in its systematic analysis of individual NSAIDs, addressing a significant gap in the literature. Moreover, this review also identifies key gaps in the literature, such as the lack of time‐to‐event data and inconsistent reporting of demographic variables like age, sex, and race.

### Limitations

All the studies included in the analyses reported that NSAID use preceded GI bleeding. However, many did not provide clear descriptions of the timing for when the bleeding event occurred after starting NSAID therapy. While Rahme *et al*. and Wan Ghazali *et al*. offered valuable time‐to‐event analyses and HR,^35^, ^40^ most studies lacked granular temporal data, making it difficult to determine the period of greatest vulnerability to GI bleeding. Additionally, most studies lacked dose–response data, limiting our understanding of how NSAID dosage influences GI bleeding risk. This omission is significant as it is believed that higher doses are often associated with an increased risk of gastrointestinal complications.^15^

Furthermore, clinical heterogeneity, particularly in terms of unreported dose and treatment duration, can affect the results. Although random effects models account for between‐study variability, the lack of detailed and consistent reporting across studies precludes more nuanced subgroup or dose–response analyses that could better explain observed differences. These limitations highlight the need for future research to focus on time‐to‐event models and incorporate dose–response analyses to better understand the temporal dynamics and dose‐related risks of GI bleeding associated with NSAID use.

Another limitation is the underrepresentation of certain NSAIDs in literature. While less commonly prescribed, drugs such as oxaprozin, sulindac, nabumetone, diflunisal, flurbiprofen, mefenamic acid, and etodolac are still used for specific conditions. Yet, data on their GI bleeding risks remain limited. Future research should first assess the current usage patterns of these NSAIDs to determine their clinical relevance and prescribing frequency. If they are still used, further studies should focus on evaluating their GI bleeding risks to provide a more comprehensive risk assessment and improve clinical decision‐making.

Finally, the literature search was conducted exclusively through PubMed. While this database includes a comprehensive range of peer‐reviewed biomedical studies, it is possible that other published reports are not included. The authors examined cited references and also reports that cited the included studies to identify other potential studies. In addition, given PubMed’s inclusion of journals in the field of clinical and pharmacoepidemiologic research, we believe that it is unlikely that relevant studies were not identified.

## CONCLUSION

This review underscores the significant variability in GI bleeding risk among individual NSAIDs, revealing a gradient from the safer profile for celecoxib to a very high risk of bleeding for ketorolac. These findings highlight the critical importance of personalized NSAID selection, particularly for high‐risk patients such as older individuals and/or those on anticoagulants. While celecoxib appears to be associated with the lowest risk of GI bleeding among the agents reviewed, its potential cardiovascular risks must also be considered in clinical decision‐making. Future research should address the gaps in dose–response relationships and time‐to‐event dynamics, and importantly, NSAIDs should not be treated as a homogenous risk for GI bleeding. Rather, the risks associated with individual NSAIDs should be analyzed separately or categorized into high‐, moderate‐, and low‐risk groups to enhance clinical decision‐making and optimize patient outcomes.

## FUNDING

This project has been supported in part by the Agency for Health Care Research and Quality (AHRQ) (Award Number U18HS029300, Implementation of DDInteract: A Shared‐decision‐Making Tool for Anticoagulant Drug–Drug INTERACTions). The content is solely the responsibility of the authors and does not represent the official views of the AHRQ.

## CONFLICT OF INTEREST

The authors declared no conflicts of interest for this work.

## AUTHOR CONTRIBUTIONS

All authors wrote the manuscript; D.C.M., A.G.L., and A.G.T., designed the research; A.G.T., A.G.L., and D.C.M. performed the research; A.G.T. analyzed the data. All authors reviewed and approved the final version of the manuscript.

## Supporting information


Data S1.
