# Supplementary material for: Nonsteroidal Anti‐Inflammatory Drugs and Risk of Gastrointestinal Bleeding: A Systematic Review and Meta‐Analysis
Source: Clin Pharmacol Ther. 2025 Sep 7;119(1):46–62. doi: 10.1002/cpt.70054 (PMC12746519; doi:10.1002/cpt.70054)
Supplement: Supplementary file 1 — Data S1. [file CPT-119-46-s001.zip › 2025-0510-s02.docx]

S2. Research term

| N |  | Research terms | Numbers |
| --- | --- | --- | --- |
| 1 | ((NSAID) AND (gastrointestinal bleeding)) OR ((NSAID) AND (gastrointestinal hemorrhage [MeSH Terms])) | (("anti inflammatory agents non steroidal"[Pharmacological Action] OR "anti inflammatory agents, non steroidal"[MeSH Terms] OR ("anti inflammatory"[All Fields] AND "agents"[All Fields] AND "non steroidal"[All Fields]) OR "non-steroidal anti-inflammatory agents"[All Fields] OR "nsaid"[All Fields] OR "nsaids"[All Fields] OR "nsaid s"[All Fields]) AND ("gastrointestinal hemorrhage"[MeSH Terms] OR ("gastrointestinal"[All Fields] AND "hemorrhage"[All Fields]) OR "gastrointestinal hemorrhage"[All Fields] OR ("gastrointestinal"[All Fields] AND "bleeding"[All Fields]) OR "gastrointestinal bleeding"[All Fields])) OR (("anti inflammatory agents non steroidal"[Pharmacological Action] OR "anti inflammatory agents, non steroidal"[MeSH Terms] OR ("anti inflammatory"[All Fields] AND "agents"[All Fields] AND "non steroidal"[All Fields]) OR "non-steroidal anti-inflammatory agents"[All Fields] OR "nsaid"[All Fields] OR "nsaids"[All Fields] OR "nsaid s"[All Fields]) AND "gastrointestinal hemorrhage"[MeSH Terms]) | 4,994 |
| 2 | ((Piroxicam) AND (gastrointestinal bleeding)) OR ((Piroxicam) AND (gastrointestinal hemorrhage [MeSH Terms])) | (("piroxicam"[MeSH Terms] OR "piroxicam"[All Fields]) AND ("gastrointestinal hemorrhage"[MeSH Terms] OR ("gastrointestinal"[All Fields] AND "hemorrhage"[All Fields]) OR "gastrointestinal hemorrhage"[All Fields] OR ("gastrointestinal"[All Fields] AND "bleeding"[All Fields]) OR "gastrointestinal bleeding"[All Fields])) OR (("piroxicam"[MeSH Terms] OR "piroxicam"[All Fields]) AND "gastrointestinal hemorrhage"[MeSH Terms]) | 99 |
| 3 | ((Naproxen ) AND (gastrointestinal bleeding)) OR ((Naproxen) AND (gastrointestinal hemorrhage [MeSH Terms])) | (("naproxen"[MeSH Terms] OR "naproxen"[All Fields] OR "naproxene"[All Fields]) AND ("gastrointestinal hemorrhage"[MeSH Terms] OR ("gastrointestinal"[All Fields] AND "hemorrhage"[All Fields]) OR "gastrointestinal hemorrhage"[All Fields] OR ("gastrointestinal"[All Fields] AND "bleeding"[All Fields]) OR "gastrointestinal bleeding"[All Fields])) OR (("naproxen"[MeSH Terms] OR "naproxen"[All Fields] OR "naproxene"[All Fields]) AND "gastrointestinal hemorrhage"[MeSH Terms]) | 232 |
| 4 | ((Meloxicam ) AND (gastrointestinal bleeding)) OR ((Meloxicam) AND (gastrointestinal hemorrhage [MeSH Terms])) | (("meloxicam"[MeSH Terms] OR "meloxicam"[All Fields]) AND ("gastrointestinal hemorrhage"[MeSH Terms] OR ("gastrointestinal"[All Fields] AND "hemorrhage"[All Fields]) OR "gastrointestinal hemorrhage"[All Fields] OR ("gastrointestinal"[All Fields] AND "bleeding"[All Fields]) OR "gastrointestinal bleeding"[All Fields])) OR (("meloxicam"[MeSH Terms] OR "meloxicam"[All Fields]) AND "gastrointestinal hemorrhage"[MeSH Terms]) | 63 |
| 5 | ((Ketorolac ) AND (gastrointestinal bleeding)) OR ((Ketorolac ) AND (gastrointestinal hemorrhage [MeSH Terms])) | (("ketorolac"[MeSH Terms] OR "ketorolac"[All Fields]) AND ("gastrointestinal hemorrhage"[MeSH Terms] OR ("gastrointestinal"[All Fields] AND "hemorrhage"[All Fields]) OR "gastrointestinal hemorrhage"[All Fields] OR ("gastrointestinal"[All Fields] AND "bleeding"[All Fields]) OR "gastrointestinal bleeding"[All Fields])) OR (("ketorolac"[MeSH Terms] OR "ketorolac"[All Fields]) AND "gastrointestinal hemorrhage"[MeSH Terms]) | 90 |
| 6 | ((Ketoprofen) AND (gastrointestinal bleeding)) OR ((Ketoprofen) AND (gastrointestinal hemorrhage [MeSH Terms])) | (("ketoprofen"[MeSH Terms] OR "ketoprofen"[All Fields] OR "ketoprofen s"[All Fields] OR "ketoprofene"[All Fields]) AND ("gastrointestinal hemorrhage"[MeSH Terms] OR ("gastrointestinal"[All Fields] AND "hemorrhage"[All Fields]) OR "gastrointestinal hemorrhage"[All Fields] OR ("gastrointestinal"[All Fields] AND "bleeding"[All Fields]) OR "gastrointestinal bleeding"[All Fields])) OR (("ketoprofen"[MeSH Terms] OR "ketoprofen"[All Fields] OR "ketoprofen s"[All Fields] OR "ketoprofene"[All Fields]) AND "gastrointestinal hemorrhage"[MeSH Terms]) | 67 |
| 7 | ((Indomethacin) AND (gastrointestinal bleeding)) OR ((Indomethacin) AND (gastrointestinal hemorrhage [MeSH Terms])) | (("indomethacin"[MeSH Terms] OR "indomethacin"[All Fields] OR "indometacin"[All Fields] OR "indomethacine"[All Fields]) AND ("gastrointestinal hemorrhage"[MeSH Terms] OR ("gastrointestinal"[All Fields] AND "hemorrhage"[All Fields]) OR "gastrointestinal hemorrhage"[All Fields] OR ("gastrointestinal"[All Fields] AND "bleeding"[All Fields]) OR "gastrointestinal bleeding"[All Fields])) OR (("indomethacin"[MeSH Terms] OR "indomethacin"[All Fields] OR "indometacin"[All Fields] OR "indomethacine"[All Fields]) AND "gastrointestinal hemorrhage"[MeSH Terms]) | 421 |
| 8 | ((Diclofenac) AND (gastrointestinal bleeding)) OR ((Diclofenac) AND (gastrointestinal hemorrhage [MeSH Terms])) | (("diclofenac"[MeSH Terms] OR "diclofenac"[All Fields]) AND ("gastrointestinal hemorrhage"[MeSH Terms] OR ("gastrointestinal"[All Fields] AND "hemorrhage"[All Fields]) OR "gastrointestinal hemorrhage"[All Fields] OR ("gastrointestinal"[All Fields] AND "bleeding"[All Fields]) OR "gastrointestinal bleeding"[All Fields])) OR (("diclofenac"[MeSH Terms] OR "diclofenac"[All Fields]) AND "gastrointestinal hemorrhage"[MeSH Terms]) | 235 |
| 9 | ((ibuprofen) AND (gastrointestinal bleeding)) OR ((ibuprofen) AND (gastrointestinal hemorrhage [MeSH Terms])) | (("ibuprofen"[MeSH Terms] OR "ibuprofen"[All Fields] OR "ibuprofen s"[All Fields] OR "ibuprofens"[All Fields]) AND ("gastrointestinal hemorrhage"[MeSH Terms] OR ("gastrointestinal"[All Fields] AND "hemorrhage"[All Fields]) OR "gastrointestinal hemorrhage"[All Fields] OR ("gastrointestinal"[All Fields] AND "bleeding"[All Fields]) OR "gastrointestinal bleeding"[All Fields])) OR (("ibuprofen"[MeSH Terms] OR "ibuprofen"[All Fields] OR "ibuprofen s"[All Fields] OR "ibuprofens"[All Fields]) AND "gastrointestinal hemorrhage"[MeSH Terms]) | 304 |
| 10 | ((celecoxib) AND (gastrointestinal bleeding)) OR ((celecoxib) AND (gastrointestinal hemorrhage [MeSH Terms])) | (("celecoxib"[MeSH Terms] OR "celecoxib"[All Fields] OR "celecoxib s"[All Fields]) AND ("gastrointestinal hemorrhage"[MeSH Terms] OR ("gastrointestinal"[All Fields] AND "hemorrhage"[All Fields]) OR "gastrointestinal hemorrhage"[All Fields] OR ("gastrointestinal"[All Fields] AND "bleeding"[All Fields]) OR "gastrointestinal bleeding"[All Fields])) OR (("celecoxib"[MeSH Terms] OR "celecoxib"[All Fields] OR "celecoxib s"[All Fields]) AND "gastrointestinal hemorrhage"[MeSH Terms]) | 206 |
|  | #1 OR #2 OR #3 OR #4 OR #5 OR #6 OR #7 OR #8 OR #9 OR #10 | (("celecoxib"[MeSH Terms] OR "celecoxib"[All Fields] OR "celecoxib s"[All Fields]) AND ("gastrointestinal hemorrhage"[MeSH Terms] OR ("gastrointestinal"[All Fields] AND "hemorrhage"[All Fields]) OR "gastrointestinal hemorrhage"[All Fields] OR ("gastrointestinal"[All Fields] AND "bleeding"[All Fields]) OR "gastrointestinal bleeding"[All Fields])) OR (("celecoxib"[MeSH Terms] OR "celecoxib"[All Fields] OR "celecoxib s"[All Fields]) AND "gastrointestinal hemorrhage"[MeSH Terms]) OR ((("ibuprofen"[MeSH Terms] OR "ibuprofen"[All Fields] OR "ibuprofen s"[All Fields] OR "ibuprofens"[All Fields]) AND ("gastrointestinal hemorrhage"[MeSH Terms] OR ("gastrointestinal"[All Fields] AND "hemorrhage"[All Fields]) OR "gastrointestinal hemorrhage"[All Fields] OR ("gastrointestinal"[All Fields] AND "bleeding"[All Fields]) OR "gastrointestinal bleeding"[All Fields])) OR (("ibuprofen"[MeSH Terms] OR "ibuprofen"[All Fields] OR "ibuprofen s"[All Fields] OR "ibuprofens"[All Fields]) AND "gastrointestinal hemorrhage"[MeSH Terms])) OR ((("diclofenac"[MeSH Terms] OR "diclofenac"[All Fields]) AND ("gastrointestinal hemorrhage"[MeSH Terms] OR ("gastrointestinal"[All Fields] AND "hemorrhage"[All Fields]) OR "gastrointestinal hemorrhage"[All Fields] OR ("gastrointestinal"[All Fields] AND "bleeding"[All Fields]) OR "gastrointestinal bleeding"[All Fields])) OR (("diclofenac"[MeSH Terms] OR "diclofenac"[All Fields]) AND "gastrointestinal hemorrhage"[MeSH Terms])) OR ((("indomethacin"[MeSH Terms] OR "indomethacin"[All Fields] OR "indometacin"[All Fields] OR "indomethacine"[All Fields]) AND ("gastrointestinal hemorrhage"[MeSH Terms] OR ("gastrointestinal"[All Fields] AND "hemorrhage"[All Fields]) OR "gastrointestinal hemorrhage"[All Fields] OR ("gastrointestinal"[All Fields] AND "bleeding"[All Fields]) OR "gastrointestinal bleeding"[All Fields])) OR (("indomethacin"[MeSH Terms] OR "indomethacin"[All Fields] OR "indometacin"[All Fields] OR "indomethacine"[All Fields]) AND "gastrointestinal hemorrhage"[MeSH Terms])) OR ((("ketoprofen"[MeSH Terms] OR "ketoprofen"[All Fields] OR "ketoprofen s"[All Fields] OR "ketoprofene"[All Fields]) AND ("gastrointestinal hemorrhage"[MeSH Terms] OR ("gastrointestinal"[All Fields] AND "hemorrhage"[All Fields]) OR "gastrointestinal hemorrhage"[All Fields] OR ("gastrointestinal"[All Fields] AND "bleeding"[All Fields]) OR "gastrointestinal bleeding"[All Fields])) OR (("ketoprofen"[MeSH Terms] OR "ketoprofen"[All Fields] OR "ketoprofen s"[All Fields] OR "ketoprofene"[All Fields]) AND "gastrointestinal hemorrhage"[MeSH Terms])) OR ((("ketorolac"[MeSH Terms] OR "ketorolac"[All Fields]) AND ("gastrointestinal hemorrhage"[MeSH Terms] OR ("gastrointestinal"[All Fields] AND "hemorrhage"[All Fields]) OR "gastrointestinal hemorrhage"[All Fields] OR ("gastrointestinal"[All Fields] AND "bleeding"[All Fields]) OR "gastrointestinal bleeding"[All Fields])) OR (("ketorolac"[MeSH Terms] OR "ketorolac"[All Fields]) AND "gastrointestinal hemorrhage"[MeSH Terms])) OR ((("meloxicam"[MeSH Terms] OR "meloxicam"[All Fields]) AND ("gastrointestinal hemorrhage"[MeSH Terms] OR ("gastrointestinal"[All Fields] AND "hemorrhage"[All Fields]) OR "gastrointestinal hemorrhage"[All Fields] OR ("gastrointestinal"[All Fields] AND "bleeding"[All Fields]) OR "gastrointestinal bleeding"[All Fields])) OR (("meloxicam"[MeSH Terms] OR "meloxicam"[All Fields]) AND "gastrointestinal hemorrhage"[MeSH Terms])) OR ((("naproxen"[MeSH Terms] OR "naproxen"[All Fields] OR "naproxene"[All Fields]) AND ("gastrointestinal hemorrhage"[MeSH Terms] OR ("gastrointestinal"[All Fields] AND "hemorrhage"[All Fields]) OR "gastrointestinal hemorrhage"[All Fields] OR ("gastrointestinal"[All Fields] AND "bleeding"[All Fields]) OR "gastrointestinal bleeding"[All Fields])) OR (("naproxen"[MeSH Terms] OR "naproxen"[All Fields] OR "naproxene"[All Fields]) AND "gastrointestinal hemorrhage"[MeSH Terms])) OR ((("piroxicam"[MeSH Terms] OR "piroxicam"[All Fields]) AND ("gastrointestinal hemorrhage"[MeSH Terms] OR ("gastrointestinal"[All Fields] AND "hemorrhage"[All Fields]) OR "gastrointestinal hemorrhage"[All Fields] OR ("gastrointestinal"[All Fields] AND "bleeding"[All Fields]) OR "gastrointestinal bleeding"[All Fields])) OR (("piroxicam"[MeSH Terms] OR "piroxicam"[All Fields]) AND "gastrointestinal hemorrhage"[MeSH Terms])) OR ((("anti inflammatory agents non steroidal"[Pharmacological Action] OR "anti inflammatory agents, non steroidal"[MeSH Terms] OR ("anti inflammatory"[All Fields] AND "agents"[All Fields] AND "non steroidal"[All Fields]) OR "non-steroidal anti-inflammatory agents"[All Fields] OR "nsaid"[All Fields] OR "nsaids"[All Fields] OR "nsaid s"[All Fields]) AND ("gastrointestinal hemorrhage"[MeSH Terms] OR ("gastrointestinal"[All Fields] AND "hemorrhage"[All Fields]) OR "gastrointestinal hemorrhage"[All Fields] OR ("gastrointestinal"[All Fields] AND "bleeding"[All Fields]) OR "gastrointestinal bleeding"[All Fields])) OR (("anti inflammatory agents non steroidal"[Pharmacological Action] OR "anti inflammatory agents, non steroidal"[MeSH Terms] OR ("anti inflammatory"[All Fields] AND "agents"[All Fields] AND "non steroidal"[All Fields]) OR "non-steroidal anti-inflammatory agents"[All Fields] OR "nsaid"[All Fields] OR "nsaids"[All Fields] OR "nsaid s"[All Fields]) AND "gastrointestinal hemorrhage"[MeSH Terms])) | 5,084 |
