# Supplementary material for: Nonsteroidal Anti‐Inflammatory Drugs and Risk of Gastrointestinal Bleeding: A Systematic Review and Meta‐Analysis
Source: Clin Pharmacol Ther. 2025 Sep 7;119(1):46–62. doi: 10.1002/cpt.70054 (PMC12746519; doi:10.1002/cpt.70054)
Supplement: Supplementary file 1 — Data S1. [file CPT-119-46-s001.zip › 2025-0510-s03.docx]

**Table S3.** Quality assessment of the included studies

**S3 A. Case-control studies Newcastle - Ottawa quality assessment**

| Case-control studies | Selection | | | | Comparability | Exposure | | | TOTAL |
| --- | --- | --- | --- | --- | --- | --- | --- | --- | --- |
|  | Case definition | Representativeness | Selection of controls | Definition of controls |  | Ascertainment | Same for cases and controls | Non-response rate |  |
| Alexander 1985 [18] | * | * | X | * | * | * | * | NA | 6 |
| Battistella 2005 [20] | * | * | X | * | ** | * | * | NA | 7 |
| Begaud 1992 [41] | * | * | X | * | ** | * | * | X | 7 |
| Blot 2000 [42] | * | * | X | * | ** | X | * | X | 6 |
| de Abajo 2013 [22] | * | * | * | * | ** | * | * | NA | 8 |
| Garcia 1994 [23] | * | * | * | * | ** | * | * | NA | 8 |
| Garcia 1998 [24] | * | * | * | * | ** | * | * | NA | 8 |
| García 2001 [25] | * | * | * | * | ** | * | * | NA | 8 |
| Gutthann 1997 [26] | * | * | * | * | ** | * | * | NA | 8 |
| Lanas 2003 [27] | * | * | * | * | ** | * | * | X | 8 |
| Lanas 2006 [28] | * | * | X | * | ** | * | * | X | 7 |
| Lanas 2015 [29] | * | * | X | * | ** | * | * | X | 7 |
| Laporte 2004 [30] | * | * | X | * | ** | * | * | X | 7 |
| Lewis 2002 [31] | * | * | X | * | ** | * | * | X | 7 |
| Nobili 1992 [33] | * | * | X | * | ** | * | * | X | 7 |
| Nørgård 2004 [34] | * | * | * | * | ** | * | * | NA | 8 |
| Sakamoto 2006 [36] | * | * | * | * | ** | * | * | X | 8 |
| Savage 1993 [37] | * | * | ***** | ***** | ** | * | * | * | 9 |
| Somerville 1986 [38] | * | * | X | * | ** | * | * | X | 7 |
| Udd 2007 [39] | * | * | X | * | ** | * | * | X | 7 |

**S3 B. Cohort studies Newcastle - Ottawa quality assessment**

| Cohort Studies | Selection | | | | Comparability | Outcome | | | TOTAL |
| --- | --- | --- | --- | --- | --- | --- | --- | --- | --- |
|  | Representative exposed | Selection nonexposed | Ascertainment of exposure | Outcome not present at start |  | Assessment of outcome | Follow-up length | Adequacy of follow-up |  |
| Anderson 2020 [19] | X | * | * | * | ** | * | * | * | 8 |
| Mamdani 2002 [32] | * | * | * | * | ** | * | * | * | 9 |
| Rahme 2007 [35] | * | * | * | * | ** | * | * | * | 9 |
| Wan Ghazali 2021 [40] | X | * | * | * | ** | * | * | * | 8 |
