# Supplementary material for: Nonsteroidal Anti‐Inflammatory Drugs and Risk of Gastrointestinal Bleeding: A Systematic Review and Meta‐Analysis
Source: Clin Pharmacol Ther. 2025 Sep 7;119(1):46–62. doi: 10.1002/cpt.70054 (PMC12746519; doi:10.1002/cpt.70054)
Supplement: Supplementary file 1 — Data S1. [file CPT-119-46-s001.zip › 2025-0510-s05.pdf]

Risk of Gastrointestinal Bleeding by Unique Nonsteroidal Anti-Inflammatory Drugs by Study Design

a) Celecoxib

Source	OR	95% CI
**StudyDesign: Cohort and Nested Case-Control**		
Battistella 2005	1.70	[0.98; 2.94]
Bhala 2013	2.22	[1.16; 4.24]
Mamdani 2002	1.00	[0.66; 1.51]
Rahme 2007	0.82	[0.66; 1.01]
Total	1.24	[0.60; 2.56]
Prediction interval		[0.29; 5.22]
Heterogeneity: χ^2_3 = 12.71 (P = .005), I^2 = 76.4%		
**StudyDesign: Case-Control**		
Lanas 2006	1.00	[0.44; 2.29]
Lanas 2015	1.20	[0.52; 2.79]
Laporte 2004	0.30	[0.03; 3.51]
Nørgård 2004	1.30	[0.65; 2.60]
Total	1.13	[0.69; 1.83]
Prediction interval		[0.55; 2.31]
Heterogeneity: χ^2_3 = 1.38 (P = .71), I^2 = 0%		
Total	1.16	[0.84; 1.61]
Prediction interval		[0.56; 2.42]

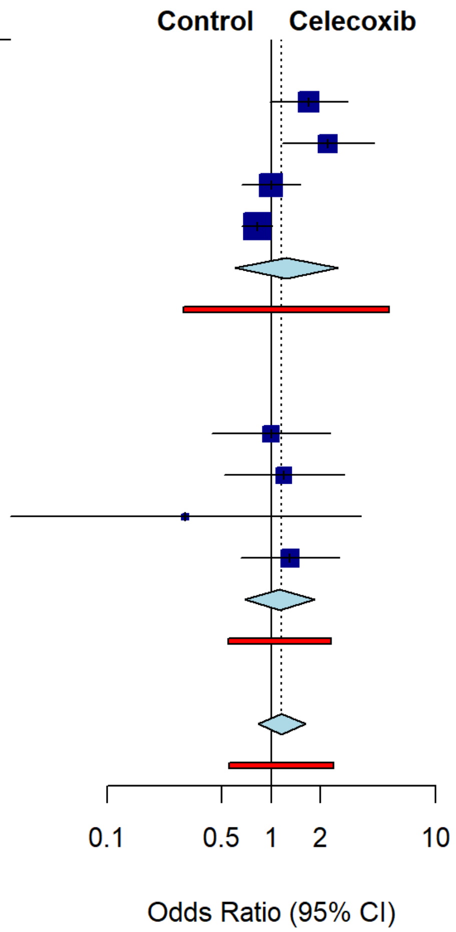

Heterogeneity: χ^2_7 = 14.42 (P = .04), I^2 = 51.4%

Test for subgroup differences: χ^2_1 = 0.12 (P = .73)

b) Diclofenac

Source	OR	95% CI
**StudyDesign: Cohort and Nested Case-Control**		
de Abajo 2013	1.32	[0.87; 1.99]
Garcia 1994	3.90	[2.32; 6.56]
García 2001	4.60	[3.62; 5.84]
Gutthann 1997	3.10	[1.84; 5.22]
Rahme 2007	1.18	[0.86; 1.62]
Wan Ghazali 2021	5.29	[2.22; 12.60]
Total	2.68	[1.36; 5.30]
Prediction interval		[0.49; 14.76]
Heterogeneity: $\chi^2_5 = 61.56$ ($P < .001$), $I^2 = 91.9\%$		
**StudyDesign: Case-Control**		
Begaud 1992	6.10	[1.31; 28.51]
Garcia 1998	2.70	[1.51; 4.83]
Lanas 2003	6.10	[3.10; 12.00]
Lanas 2006	3.10	[2.29; 4.19]
Lanas 2015	3.50	[1.80; 6.80]
Laporte 2004	3.70	[2.57; 5.33]
Lewis 2002	4.90	[3.34; 7.19]
Nobili 1992	5.10	[2.78; 9.34]
Savage 1993	3.30	[1.59; 6.85]
Sakamoto 2006	10.90	[2.48; 47.96]
Total	3.90	[3.17; 4.79]
Prediction interval		[2.77; 5.48]
Heterogeneity: $\chi^2_9 = 10.08$ ($P = .34$), $I^2 = 10.7\%$		
Total	3.42	[2.58; 4.53]
Prediction interval		[1.25; 9.36]

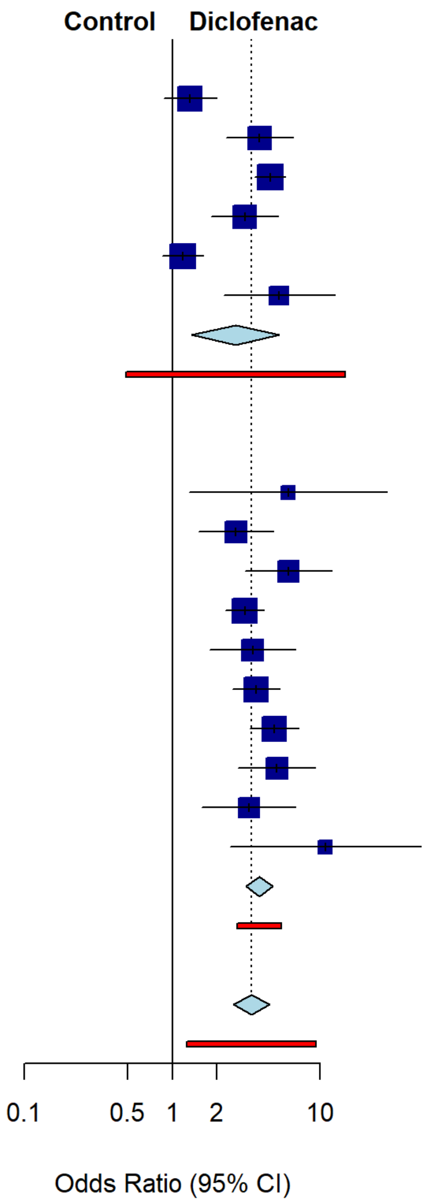

Heterogeneity: $\chi^2_{15} = 81.57$ ($P < .001$), $I^2 = 81.6\%$
Test for subgroup differences: $\chi^2_1 = 1.78$ ($P = .18$)

c) Ibuprofen

Source	OR	95% CI
**StudyDesign: Cohort and Nested Case-Control**		
de Abajo 2013	1.33	[0.94; 1.89]
Garcia 1994	2.90	[1.69; 4.97]
García 2001	2.50	[1.87; 3.34]
Gutthann 1997	2.10	[1.10; 4.00]
Rahme 2007	1.11	[0.57; 2.18]
Total	1.90	[1.15; 3.12]
Prediction interval		[0.69; 5.23]
Heterogeneity: $\chi^2_4 = 12.22$ (P = .02), $I^2 = 67.3\%$		
**StudyDesign: Case-Control**		
Alexander 1985	8.62	[4.64; 16.02]
Bolt 2000	2.40	[1.49; 3.87]
Garcia 1998	2.10	[0.61; 7.22]
Lanas 2006	4.10	[3.14; 5.36]
Lanas 2015	1.40	[0.99; 1.98]
Laporte 2004	3.10	[1.98; 4.85]
Lewis 2002	1.70	[1.13; 2.56]
Nobili 1992	3.50	[1.20; 10.20]
Savage 1993	1.90	[0.53; 6.85]
Somerville 1986	1.15	[0.51; 2.60]
Udd 2007	1.86	[0.52; 6.62]
Total	2.52	[1.70; 3.74]
Prediction interval		[0.75; 8.43]
Heterogeneity: $\chi^2_{10} = 47.24$ (P < .001), $I^2 = 78.8\%$		
Total	2.28	[1.71; 3.03]
Prediction interval		[0.83; 6.27]

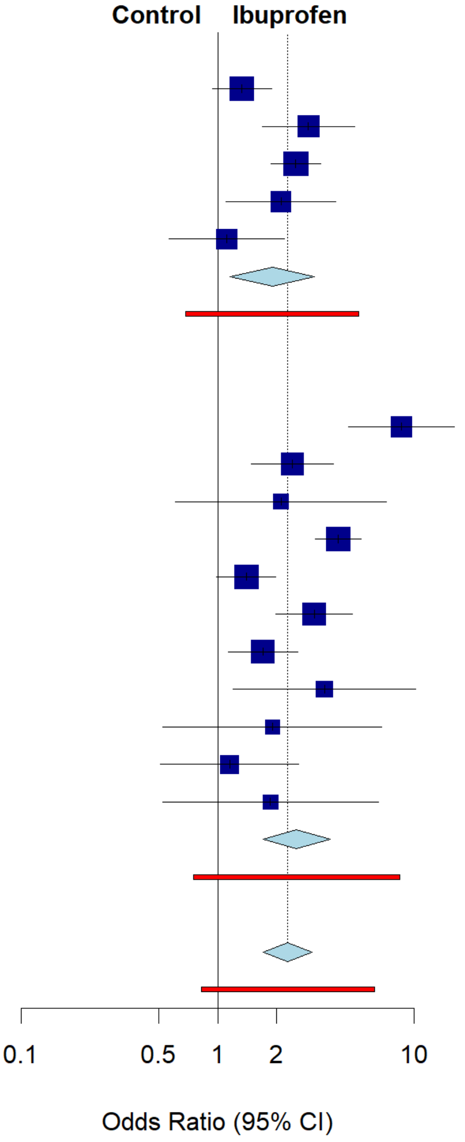

Heterogeneity: $\chi^2_{15} = 65.48$ (P < .001), $I^2 = 77.1\%$

Test for subgroup differences: $\chi^2_1 = 1.26$ (P = .26)

d) Indomethacin

Source	OR	95% CI
**StudyDesign: Cohort and Nested Case-Control**		
Garcia 1994	6.30	[3.28; 12.11]
García 2001	5.20	[3.23; 8.37]
Gutthann 1997	4.60	[3.48; 6.07]
Total	4.91	[3.57; 6.74]
Prediction interval		[2.99; 8.05]
Heterogeneity: χ^2_2 = 0.83 (P = .66), I^2 = 0%		
**StudyDesign: Case-Control**		
Alexander 1985	6.36	[2.80; 14.46]
Garcia 1998	5.50	[1.60; 18.90]
Lanas 2006	9.00	[3.91; 20.73]
Laporte 2004	10.00	[4.41; 22.66]
Lewis 2002	6.00	[3.60; 10.00]
Nobili 1992	8.30	[1.71; 40.21]
Savage 1993	13.90	[3.32; 58.17]
Somerville 1986	2.63	[1.14; 6.06]
Total	6.44	[4.39; 9.43]
Prediction interval		[3.90; 10.62]
Heterogeneity: χ^2_7 = 7.5 (P = .38), I^2 = 6.6%		
Total	5.52	[4.43; 6.86]
Prediction interval		[4.07; 7.47]

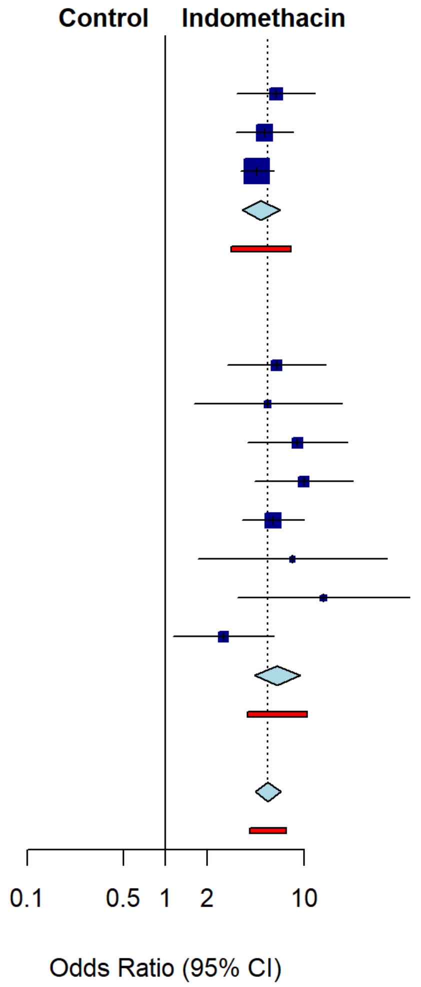

Heterogeneity: χ^2_{10} = 10.27 (P = .42), I^2 = 2.6%

Test for subgroup differences: χ^2_1 = 2.34 (P = .13)

e) Ketoprofen

Source	OR	95% CI
**StudyDesign: Cohort and Nested Case-Control**		
de Abajo 2013	3.79	[1.70; 8.45]
Garcia 1994	5.40	[2.59; 11.26]
García 2001	3.30	[1.87; 5.82]
Gutthann 1997	4.90	[2.44; 9.85]
Total	4.14	[2.84; 6.03]
Prediction interval		[2.38; 7.20]
Heterogeneity: χ^2_3 = 1.39 (P = .71), I^2 = 0%		
**StudyDesign: Case-Control**		
Alexander 1985	6.45	[0.66; 62.85]
Garcia 1998	3.20	[0.88; 11.64]
Lanas 2006	8.60	[2.52; 29.39]
Lanas 2015	1.90	[0.63; 5.75]
Laporte 2004	4.86	[2.70; 8.75]
Lewis 2002	34.90	[12.66; 96.20]
Savage 1993	2.40	[0.99; 5.83]
Udd 2007	6.15	[1.33; 28.38]
Total	5.41	[2.44; 12.00]
Prediction interval		[0.69; 42.59]
Heterogeneity: χ^2_7 = 20.95 (P = .004), I^2 = 66.6%		
Total	4.84	[3.05; 7.68]
Prediction interval		[1.42; 16.51]

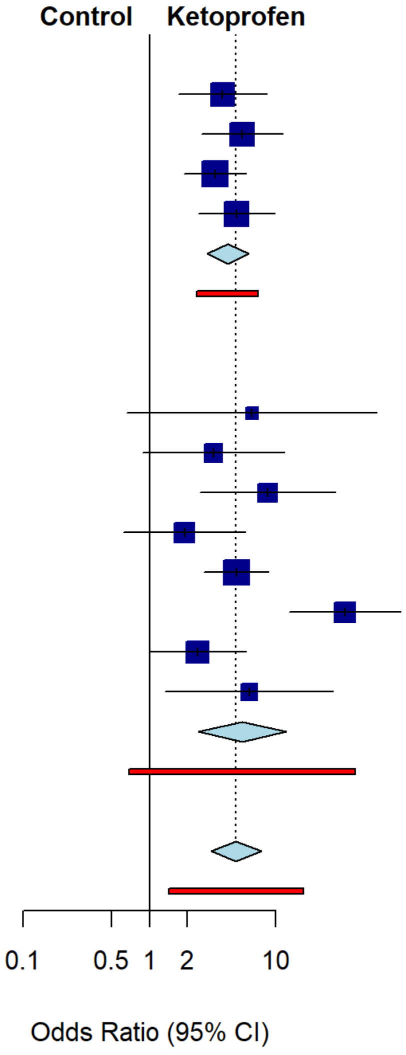

Heterogeneity: χ^2_{11} = 23.17 (P = .02), I^2 = 52.5%

Test for subgroup differences: χ^2_1 = 0.57 (P = .45)

f) Ketorolac

Source	OR	95% CI
**StudyDesign: Cohort and Nested Case-Control**		
Anderson 2020	15.21	[0.72; 320.14]
**StudyDesign: Case-Control**		
Garcia 1998	24.70	[9.60; 63.52]
Lanas 2003	24.80	[3.13; 196.69]
Lanas 2006	14.40	[5.20; 39.89]
Laporte 2004	24.70	[7.96; 76.63]
Total	20.89	[13.20; 33.07]
Prediction interval		[8.30; 52.56]
Heterogeneity: $\chi^2_3 = 0.74$ (P = .86), $I^2 = 0\%$		
Total	20.67	[14.56; 29.34]
Prediction interval		[9.37; 45.60]

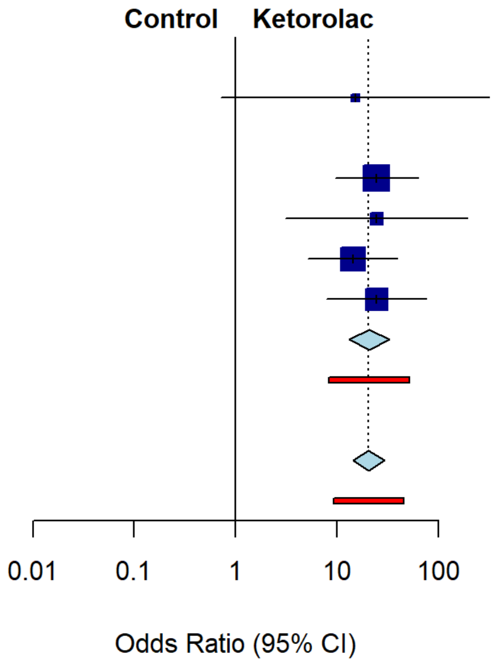

Heterogeneity: $\chi^2_4 = 0.78$ (P = .94), $I^2 = 0.0\%$

Test for subgroup differences: $\chi^2_1 = 0.04$ (P = .84)

g) Meloxicam

Source	OR	95% CI
**StudyDesign: Cohort and Nested Case-Control**		
García 2001	3.80	[0.82; 17.62]
**StudyDesign: Case-Control**		
Lanas 2006	9.80	[4.02; 23.90]
Laporte 2004	5.70	[2.18; 14.88]
Total	7.62	[0.25; 236.25]
Prediction interval		[0.11; 526.55]
Heterogeneity: $\chi^2_1 = 0.66$ (P = .42), I^2 = 0%		
Total	6.85	[2.34; 20.07]
Prediction interval		[1.83; 25.63]

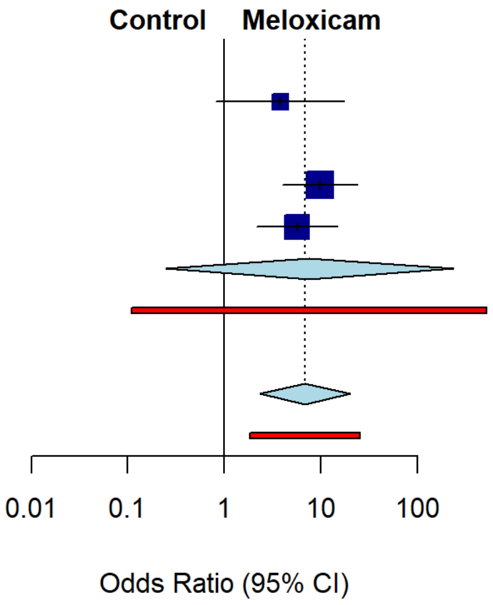

Heterogeneity: $\chi^2_2 = 1.33$ (P = .51), I^2 = 0.0%

Test for subgroup differences: $\chi^2_1 = 0.71$ (P = .40)

h) Naproxen

Source	OR	95% CI
**StudyDesign: Cohort and Nested Case-Control**		
de Abajo 2013	2.14	[1.03; 4.46]
Garcia 1994	3.10	[1.66; 5.78]
García 2001	4.00	[2.78; 5.76]
Gutthann 1997	3.50	[2.77; 4.43]
Rahme 2007	2.75	[2.05; 3.69]
Total	3.25	[2.58; 4.08]
Prediction interval		[2.44; 4.32]
Heterogeneity: $\chi^2_4 = 4.14$ (P = .39), $I^2 = 3.4\%$		
**StudyDesign: Case-Control**		
Alexander 1985	3.25	[0.72; 14.69]
Garcia 1998	4.30	[1.63; 11.38]
Lanas 2003	7.00	[2.51; 19.55]
Lanas 2006	7.30	[4.69; 11.37]
Lanas 2015	1.70	[0.77; 3.75]
Laporte 2004	10.00	[5.69; 17.57]
Lewis 2002	9.10	[6.02; 13.75]
Nobili 1992	3.50	[1.20; 10.20]
Savage 1993	5.10	[2.37; 10.97]
Somerville 1986	4.74	[1.35; 16.64]
Total	5.52	[3.70; 8.25]
Prediction interval		[1.91; 16.01]
Heterogeneity: $\chi^2_9 = 19.51$ (P = .02), $I^2 = 53.9\%$		
Total	4.31	[3.22; 5.77]
Prediction interval		[1.60; 11.59]

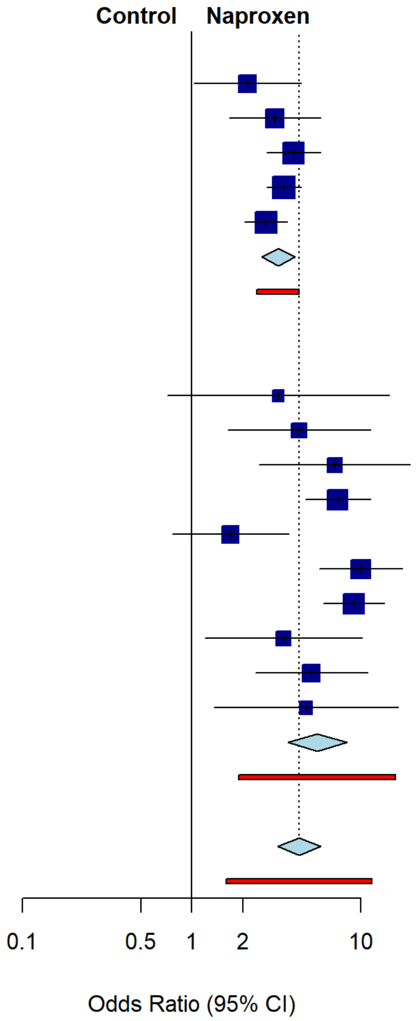

Heterogeneity: $\chi^2_{14} = 49.43$ (P < .001), $I^2 = 71.7\%$

Test for subgroup differences: $\chi^2_1 = 7.39$ (P = .007)

i) Piroxicam

Source	OR	95% CI
**StudyDesign: Cohort and Nested Case-Control**		
de Abajo 2013	4.50	[2.37; 8.56]
Garcia 1994	18.00	[8.19; 39.56]
García 2001	6.20	[3.75; 10.24]
Gutthann 1997	4.50	[3.53; 5.74]
Total	6.46	[2.44; 17.11]
Prediction interval		[1.02; 40.78]
Heterogeneity: χ^2_3 = 11.57 (P = .009), I^2 = 74.1%		
**StudyDesign: Case-Control**		
Alexander 1985	39.21	[3.52; 436.58]
Begaud 1992	70.11	[7.79; 630.91]
Garcia 1998	9.50	[6.52; 13.84]
Lanas 2003	15.40	[6.40; 37.08]
Lanas 2006	12.60	[7.81; 20.33]
Laporte 2004	15.50	[9.96; 24.11]
Lewis 2002	13.10	[7.89; 21.76]
Nobili 1992	2.50	[0.59; 10.51]
Savage 1993	6.40	[2.77; 14.81]
Somerville 1986	7.66	[1.74; 33.73]
Total	11.63	[8.58; 15.78]
Prediction interval		[8.16; 16.57]
Heterogeneity: χ^2_9 = 13.64 (P = .14), I^2 = 34%		
Total	9.24	[6.34; 13.45]
Prediction interval		[3.17; 26.94]

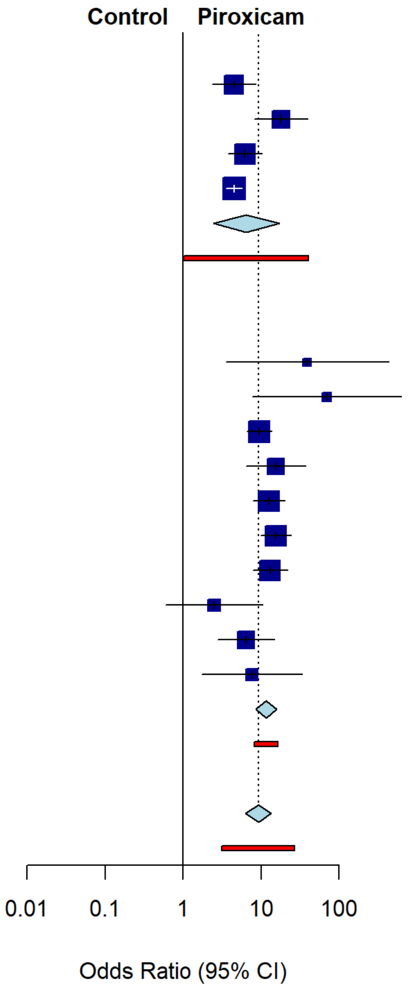

Heterogeneity: χ^2_{13} = 56.18 (P < .001), I^2 = 76.9%

Test for subgroup differences: χ^2_1 = 3.10 (P = .08)
