# Supplementary figures and images for: Nonsteroidal Anti‐Inflammatory Drugs and Risk of Gastrointestinal Bleeding: A Systematic Review and Meta‐Analysis
Source: Clin Pharmacol Ther. 2025 Sep 7;119(1):46–62. doi: 10.1002/cpt.70054 (PMC12746519; doi:10.1002/cpt.70054)

1. Celecoxib Forest Plot after removing Bhala 2013


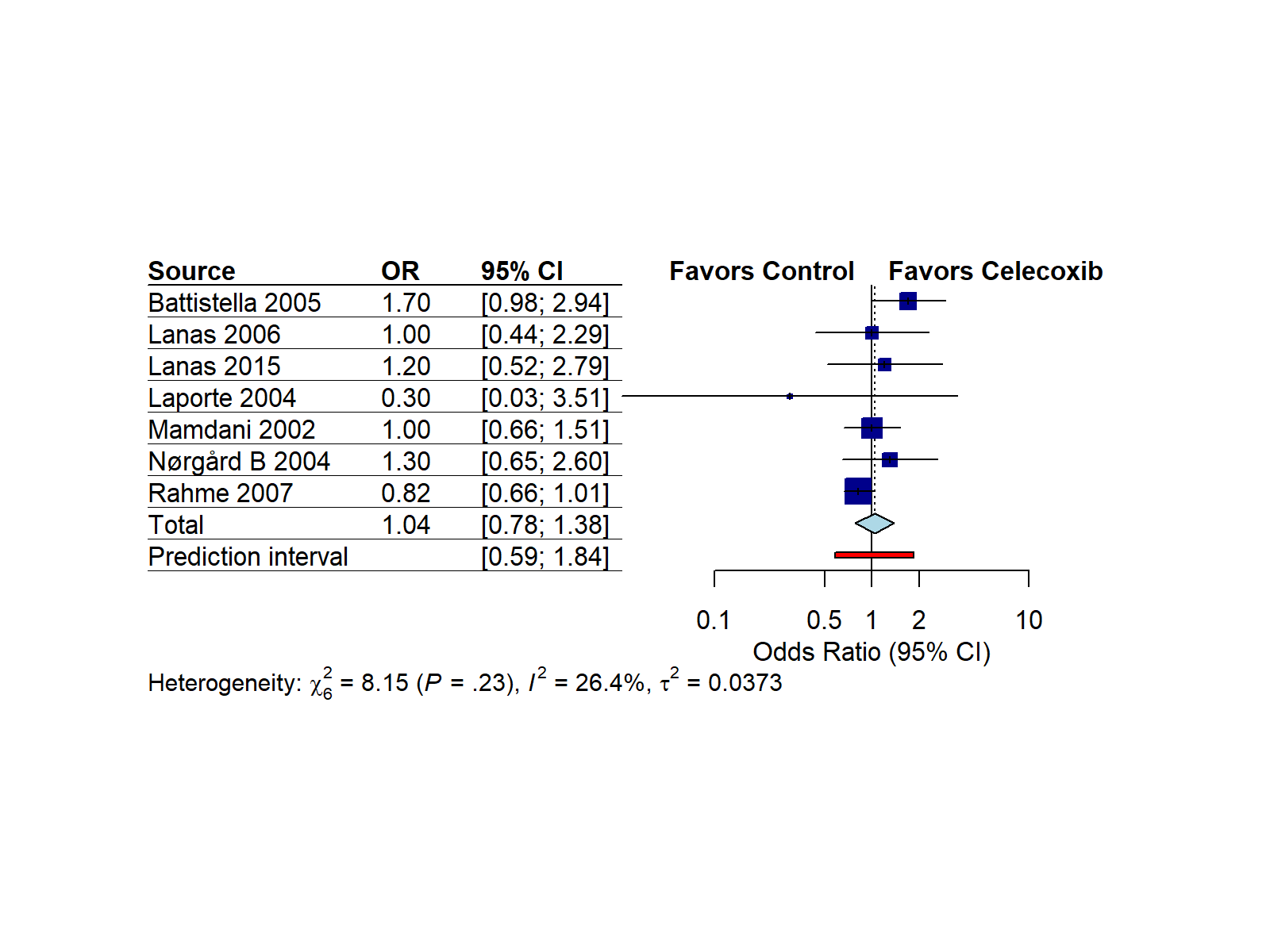

Supplement: Supplementary file 1 — Data S1. [file CPT-119-46-s001.zip › 2025-0510-s04.docx]
